# Beyond heartbeats: re-thinking interoception in autism through virtual reality and computational modelling

**DOI:** 10.3389/fncir.2026.1934104

**Published:** 2026-10-02

**Authors:** Sarune Savickaite, Ruichen Li, Yukie Nagai

**Affiliations:** 1 Department of Psychology, University of Exeter, Exeter, United Kingdom; 2 International Research Center for Neurointelligence (WPI-IRCN), The University of Tokyo, Tokyo, Japan

**Keywords:** active inference, autism, computational modelling, interoception, lived experience, predictive processing, virtual reality

## Abstract

Interoception, the sensing and interpretation of internal bodily signals, is increasingly invoked to explain heterogeneity in autism, yet empirical findings remain inconsistent. A major reason is methodological: the field remains cardio-centric and relies heavily on heartbeat counting tasks and trait questionnaires, which are vulnerable to cognitive strategies, attention, expectancy effects, and confounds such as alexithymia and anxiety. Autism research further indicates that interoception is multidimensional (accuracy, sensibility, metacognitive awareness) and developmentally dynamic, with evidence for dissociations across components and compensation across the lifespan. We argue that progress requires reframing interoception as an inference problem: the brain estimates hidden physiological states from noisy sensory evidence under context-dependent precision weighting (predictive processing/active inference). Building on this framework, we propose a participatory, multimodal research program for autism that integrates (i) lived experience as hypothesis-shaping data, (ii) virtual reality (VR) as an experimental testbed to manipulate embodiment, sensory load, agency cues, and feedback, and (iii) computational physiological modelling to estimate latent cardiovascular–respiratory and autonomic-control parameters, which could subsequently constrain separately specified models of perceptual inference.

## Introduction

Interoception encompasses the sensing, integration and interpretation of signals originating within the body, including cardiac, respiratory, gastrointestinal and nociceptive signals. A widely used multidimensional framework distinguishes interoceptive accuracy, referring to objective performance in detecting internal signals; interoceptive sensibility, referring to subjective beliefs and self-reported tendencies; and interoceptive awareness, referring to metacognitive correspondence between objective performance and confidence (Garfinkel et al., 2015).

Neurodiversity refers to the inherent variation of human minds and nervous systems, and that these variations (e.g., autistic, ADHD, dyslexic) are part of normal human diversity rather than defects that require “correction.” The notion was first articulated within autistic self-advocacy societies and popularized in Judy Singer’s, 1998 thesis; current scholarship views neurodiversity as both a descriptive reality and a civil-rights-oriented approach to disability (Singer, 1998, 2017; Dwyer, 2022; Botha et al., 2024).

Neurodivergence refers broadly to individuals whose cognitive, sensory, attentional or affective processing differs from culturally predominant neurocognitive patterns. While not a diagnostic word, it is commonly used in academia and practice to describe lived processing differences that frequently overlap across categories (e.g., autism and ADHD) (Dwyer, 2022; National Autistic Society, n.d.). Within this framework, differences in interoceptive processing should not be treated as isolated “deficits” but as situated within broader patterns of neurocognitive diversity. Importantly, interoception has been increasingly implicated as a domain in which neurodivergent experiences diverge from normative assumptions embedded in standard experimental paradigms.

Autism is a neurodevelopmental condition defined by the DSM-5-TR as (a) persistent differences in social communication and interaction across contexts, and (b) restricted, repetitive patterns of behavior, interests, or activities, with onset in early childhood and significant heterogeneity in presentation and support needs (American Psychiatric Association, 2022). Among neurodivergent groups, autism has received disproportionate attention in interoception research, often serving as the primary basis for claims about atypical bodily awareness. However, much of this literature relies on paradigms that are poorly suited to the sensory, cognitive, and predictive differences characteristic of autistic experience. As such, autism provides a particularly instructive context for evaluating both the limitations of traditional interoceptive tasks and the potential of emerging approaches–such as immersive virtual environments and computational modelling–to better capture embodied experience.

The central contribution of this Perspective is therefore not the proposal that VR, computational modelling, or participatory research should independently be added to interoception research. Rather, we propose a multilevel framework in which each addresses a different component of the same inferential problem. Lived experience identifies which bodily signals and contexts are meaningful and generates hypotheses about when interoceptive access changes. VR provides controlled perturbations of those contexts, including embodiment, sensory load, social cues, agency, and physiological feedback. Peripheral physiological models characterize the cardiovascular, respiratory, and autonomic dynamics that generate the bodily evidence available to the nervous system, while separately specified perceptual-inference models can test hypotheses about how that evidence is integrated with prior expectations and contextual information. Behavioral performance, state-level confidence, subjective experience, and physiological measurements provide complementary observations against which these hypotheses can be evaluated. The novelty of the framework therefore lies in connecting these levels within a participatory and experimentally falsifiable programme for studying heterogeneity in autistic interoception.

## Interoceptive processing in autism: evidence for heterogeneity rather than deficit

Interoception has emerged as a useful lens for understanding autistic experience, but the data is conflicting and methodologically diverse. A significant thread is the “alexithymia hypothesis,” which states that numerous socio-emotional challenges associated with autism are better explained by co-occurring alexithymia (difficulty identifying/describing emotions). Canonically, Shah et al. (2016) demonstrated in two tests that interoceptive dysfunction tracked alexithymia rather than autism *per se*, a finding anticipated by Brewer et al. (2015) and echoed in Livingston and Livingston’s (2016) discussion. This is important for a study design because without quantifying alexithymia, group differences may be misattributed.

In addition, Garfinkel et al. (2016) argued that interoception is multidimensional (accuracy, sensibility, awareness/metacognition) and that mismatch between dimensions (e.g., high confidence/attention with low objective accuracy) correlates with anxiety in autistic adults - an idea that was later used in interoceptive training trials (e.g., ADIE; Quadt et al., 2021). Nicholson et al. (2019) found that autistic children had lower cardiac interoceptive accuracy, which appeared to resolve or be compensated for in adulthood, undermining “unitary deficit” theories and emphasizing trajectories and compensation.

Neurocognitive descriptions go beyond behavior. Butera et al. (2023a,b) link alexithymia and interoception to empathy and functional connectivity during socio-emotional processing in autistic youth, emphasizing interhemispheric patterns and implying that interoceptive/affective factors modulate social cognition differently across individuals. Broader systems-level suggestions (e.g., Leisman et al., 2023) place interoception inside distributed circuits (PFC-basal ganglia-thalamus-cerebellum), which is consistent with embodied models of cognition.

Self-report scales (e.g., BAQ; thirst-specific measures; the Interoception Sensory Questionnaire) are extensively employed, but psychometrics are developing. Suzman et al. (2021) refined the ISQ structure in autistic populations, whereas Itoi et al. (2022) validated the ISQ-J, emphasizing cross-cultural features and the need of distinguishing between subjective difficulty and correctness. Intervention work is emerging: pilot classroom programs such as the Interoception Curriculum show increases in interview-based interoceptive awareness for autistic children, albeit in small samples with few controls (Hample et al., 2020, pilot).

The consensus among scope reviews is to proceed with caution. Hatfield et al. (2019) emphasize that atypical interoceptive processing is increasingly reported, but findings may be due to alexithymia and measurement artefacts; Loureiro et al. (2024) identify heterogeneity in tasks (e.g., heartbeat counting vs. discrimination), populations (age, IQ, co-occurring conditions), and analytic frameworks as sources of inconsistency. A recent systematic review/meta-analysis found mixed effects across accuracy, sensibility, and awareness, emphasizing the importance of separating constructs and controlling for alexithymia and anxiety (Klein et al., 2025).

## Heterogeneity as a person-level component

Heterogeneity should be incorporated into experimental and computational designs rather than treated solely as unexplained variance. In addition to alexithymia and anxiety, relevant person-level variables include age and developmental stage, intellectual and language abilities, co-occurring neurodevelopmental or mental-health conditions, medication, sex and gender, physical fitness and autonomic status, sensory profile, communication preferences, and previous experience or tolerance of immersive technology. These factors may operate at different levels of the proposed framework. Physical fitness, medication and autonomic status may alter the physiological signal available for interoception; sensory profile and attentional style may affect the salience or reliability of competing internal and external information; while communication preferences may affect how subjective interoceptive experience can be captured.

Sensory processing warrants particular attention in VR. Hyper- or hyporesponsivity may influence physiological arousal, attentional allocation, presence, susceptibility to visual–vestibular conflict, simulator discomfort and willingness to remain within an immersive task. Variation in sensory profile should therefore be measured as a potential moderator rather than assumed to be background noise. Recent work demonstrating associations between sensory profiles and autistic characteristics in children further supports treating sensory processing as an individual-difference dimension within autism research (Levante et al., 2026). The objective is not to statistically control away autistic heterogeneity, but to determine which dimensions explain meaningful differences in the relationship between bodily signals, perceptual judgements and context.

## Neural circuits of interoceptive inference

Interoceptive inference is implemented across a distributed hierarchy rather than within a single cortical region. Visceral information reaches the central nervous system through multiple ascending pathways. Vagal and glossopharyngeal afferents carrying information from cardiovascular, respiratory, gastrointestinal and other visceral systems converge prominently within the nucleus of the solitary tract (NTS), while spinal visceral afferents enter the dorsal horn and ascend through pathways including the lamina I spinothalamic system. At the brainstem level, the NTS interacts with the parabrachial nucleus, periaqueductal grey, ventrolateral medulla and hypothalamic structures, providing an early interface between representation of bodily state and autonomic regulation (Critchley and Harrison, 2013). These pathways therefore already involve integration rather than the passive transmission of an isolated cardiac or respiratory signal.

Ascending viscerosensory information is subsequently integrated through thalamic and subcortical pathways with cortical representations involving the insular and cingulate cortices. The insula is particularly important but should not be conceptualized as a single interoceptive “center.” A posterior-to-anterior organization has been proposed in which more posterior insular regions support relatively primary representations of bodily state, while anterior insular cortex integrates interoceptive information with motivational, affective and exteroceptive information to contribute to consciously accessible bodily experience (Critchley and Harrison, 2013; Seth, 2013). The anterior insula is closely functionally connected with the anterior cingulate cortex within the salience network, while both interact with regions including the amygdala, orbitofrontal cortex, periaqueductal grey and brainstem autonomic nuclei. This architecture therefore links visceral sensing, salience detection, attention and autonomic control rather than treating interoception as an exclusively sensory process.

Human neuroimaging provides empirical support for this distributed account. During heartbeat detection, Critchley et al. (2004) observed increased activity in bilateral insular, somatomotor and anterior cingulate regions, with activity in right anterior insular/opercular cortex predicting objective interoceptive accuracy. Importantly, anterior insular responses were sensitive not simply to bodily stimulation but to the relationship between attentional focus and mismatched cardiac feedback, suggesting that neural responses to interoceptive signals depend on how those signals are attended to and interpreted. This distinction is particularly relevant to autism research, where attention, anxiety, alexithymia and sensory context may influence both access to bodily information and beliefs about that information.

Predictive-processing accounts provide one possible functional interpretation of this hierarchy. Rather than ascending visceral signals determining perception directly, interoceptive inference can be conceptualized as an interaction between bottom-up bodily evidence and descending predictions about its likely causes. Seth (2013) proposed that the anterior insula participates within an extended autonomic and salience network in comparing predicted and incoming interoceptive signals, while the influence of resulting prediction errors depends on their estimated precision. Within predictive coding, precision can be understood as the estimated reliability of a signal and may be implemented partly through modulation of the gain of prediction-error signaling populations. Attention can therefore alter interoceptive perception by changing how strongly ascending evidence influences higher-level inference. Descending predictions may also contribute to autonomic control through interactions with brainstem regulatory circuits, linking perception and physiological regulation within a recurrent brain–body loop.

This neural-circuit account is important for the framework proposed here because the different methodological components address different levels of this hierarchy. Physiological models characterize the peripheral cardiovascular, respiratory and autonomic dynamics that generate bodily evidence; they do not directly estimate neural prediction errors or cortical precision weighting. VR instead provides experimentally controlled changes to the exteroceptive, multisensory, social and attentional context in which that bodily evidence is interpreted. Behavioral and subjective measures index outcomes of these interactions, while future neuroimaging or neurophysiological measurements could test how VR manipulations influence central representations and network dynamics. The proposed framework therefore treats interoception as a multilevel brain–body process in which peripheral physiology constrains the signals available for inference, while distributed neural circuits determine how those signals are integrated with context, expectations and action.

## Interoception as multilevel inference: a framework integrating lived experience, VR, and computational modelling

Interoception is deeply personal, context-dependent, and frequently ineffable, which makes it both fascinating and difficult to quantify. We are seeing more lived-experience work from neurodivergent communities, such as autistic teenagers and adults. Trevisan et al. (2021) examined first-person experiences and found patterns of hyposensitivity to hunger/thirst and hyper-attention to anxiety-related sensations (e.g., respiration, heartbeat), which mirrored clinical observations. Newer qualitative research with autistic teens connects interoceptive experiences to anxiety and daily management (Adams et al., 2025). However, these rich narratives seldom map cleanly onto typical lab tasks: many quantitative research use self-report composites or cardiac heartbeat counts, both of which have acknowledged drawbacks (task compliance, expectation effects, confounding by attention/fitness/anxiety). This creates an interpretation gap between what people say they have experienced and what our present techniques can detect.

Interoception can be formally understood as a hierarchical inference process in which the brain estimates hidden physiological states based on noisy sensory inputs, prior beliefs, and contextual information (Barrett and Simmons, 2015; Allen et al., 2022). Predictive-processing and active-inference accounts propose that interoceptive perception arises from ongoing comparisons between top–down predictions and bottom–up bodily signals, with perceived sensations shaped by the relative weighting—or precision—assigned to each source of information (Feldman and Friston, 2010; Ainley et al., 2016). This framework provides a unifying structure for understanding the heterogeneity of interoception in autism, as differences in bodily awareness, alexithymia, anxiety, attentional style, or sensory load (Lawson et al., 2014; Van de Cruys et al., 2017; Friston et al., 2013) can each be recast as alterations in priors, precision weighting, or update dynamics within the inference system.

However, current empirical approaches fall short because they operationalize interoception only at the behavioral level (e.g., counting heartbeats) or the subjective level (e.g., questionnaires), leaving the latent inferential processes inaccessible. To overcome this, lived experiences, VR manipulations, and computational modelling should be treated as complementary levels of evidence.

There is ample room to (1) tighten quantitative frameworks - separating accuracy, sensibility, and metacognition; routinely measuring alexithymia and anxiety; diversifying interoceptive modalities (cardiac, respiratory, gastrointestinal, nociceptive) beyond heartbeat tasks; and improving psychometrics, and (2) incorporate lived experience into our methods so that findings are meaningful to autistic participants.

The framework developed in this Perspective therefore integrates three complementary methodological components within a common neural and inferential account. First, lived experience and participatory research identify meaningful bodily signals, contexts and sources of heterogeneity and shape the hypotheses to be tested. Second, VR provides experimentally controlled manipulations of embodiment, sensory evidence, attentional load, social context, agency and physiological feedback while supporting simultaneous behavioral and physiological measurement. Third, computational approaches characterize different levels of the resulting system: mechanistic physiological models estimate latent peripheral dynamics, separately specified perceptual-inference models formalize hypotheses about interpretation of bodily evidence, and machine-learning methods may support multimodal estimation and adaptive experimentation. These components are connected through distributed brain–body circuits rather than representing interchangeable measures of interoception.

## Virtual reality as a testbed for interoceptive inference

Virtual reality (VR) is defined as interactive, computer-generated environments that provide sensorimotor conditions similar to the physical world and can elicit a convincing “sense of being there” (presence). Presence is often defined as the mix of location illusion (feeling like you are in the virtual area) and plausibility (feeling like events are actually happening), both of which influence how people experience and act in VR (Slater et al., 2022). Embodiment is a unique feature of VR: from a first-person perspective, users can feel ownership and agency over a virtual body or limb, which influences perception, affect, and physiology (Gonzalez-Franco and Peck, 2018; Peck and Gonzalez-Franco, 2021). These characteristics make VR an excellent testbed for interoception: it can carefully regulate exteroceptive input (what you see, hear, and feel) while also investigating how internal signals are perceived and how context shapes that perception. Building on predictive-processing accounts, some have argued that presence should be expanded beyond “place illusion” to include a felt, interoceptive being dimension–that is, presence emerges when the brain’s predictions about both world-related and body-related causes are correct (Seth et al., 2012).

Across the small and heterogeneous literature identified here, VR has primarily been employed as a controlled scaffolding tool to (a) render or perturb bodily signals, (b) orient attention towards or away from internal signals, and (c) combine physiological recording with behavioral or subjective measures. This builds on a broader experimental tradition in interoception research that relies on behavioral accuracy paradigms (e.g., heartbeat detection tasks) alongside physiological recording to index the correspondence between perceived and actual bodily states (e.g., Garfinkel et al., 2015). Such work highlights that interoception is not a unitary construct, but comprises dissociable dimensions (e.g., accuracy, sensibility, awareness), providing a conceptual foundation for VR-based extensions.

## Identification of relevant VR–interoception literature

As this Perspective Article does not constitute a systematic review, a targeted literature search was undertaken to identify research directly relevant to the use of virtual reality for studying interoception. Searches were conducted on 20 August 2026 using PubMed and Google Scholar. The search terms “interoception,” “virtual reality,” and “autism” were entered in combination in both databases. The purpose of the search was to identify studies examining interoceptive processes within immersive or virtual environments, with particular attention to their potential relevance for autism research.

The PubMed search identified four records, which were screened for relevance based on title and abstract; two were retained as directly relevant to the present discussion. In Google Scholar, eight records were identified for screening, of which seven were retained following title screening and assessment of relevance. Records were considered relevant where they examined interoception, bodily awareness, or closely related embodied processes in conjunction with VR or immersive environments. Given the limited literature directly combining autism, interoception, and VR, studies involving other populations were retained where their methodology or findings could inform the development of VR-based interoception paradigms for autism.

The search was intended to improve transparency and identify directly relevant methodological evidence rather than to constitute a systematic review. Accordingly, the resulting literature includes different forms of evidence, including peer-reviewed empirical studies, a study protocol, and postgraduate research. These distinctions are identified in Table 1.

**TABLE 1:** Summary of studies examining interoception in virtual or immersive environments.

Study	Population	VR manipulation	Interoceptive modality	Main findings
Breuninger et al., 2017	Adults with agoraphobia (*n* = 21) and healthy controls (*n* = 27)	VR anxiety-induction/stressor used to elicit physiological and subjective responses	Cardiac/autonomic: heart rate, HRV and subjective perception of physiological arousal	Participants with agoraphobia showed blunted cardiac/autonomic reactivity despite heightened subjective experience during VR anxiety induction, demonstrating a dissociation between physiological responses and perceived bodily arousal.
El Ali et al., 2023	Adults (*N* = 50)	Screen versus VR cardiac-recognition task; displayed HR manipulated by ± 15%, ± 30% or not altered; audio, visual and audiovisual feedback	Cardiac interoceptive accuracy and awareness	VR itself did not significantly change cardiac interoceptive accuracy relative to screen presentation. Audio feedback increased interoceptive awareness, while greater VR presence was negatively associated with cardiac accuracy, suggesting immersion may compete with attention to internal signals.
Sebri et al., 2023	Breast cancer survivors; planned *N* = 40 (study protocol)	Embodied VR intervention using a female avatar with visual and auditory bodily stimulation; compared with neutral VR relaxation	Multidimensional interoceptive awareness; HR and HRV also recorded	Protocol proposes that embodied VR may improve awareness of internal sensations, body image and emotional wellbeing. As a protocol, it does not yet provide empirical outcome evidence.
Torres et al., 2024	Adults with cervical spinal cord injury and non-injured controls	VR/mental imagery combined with breathing exercises, guided interoceptive attention and autonomic biofeedback	Cardiovascular/autonomic: blood pressure, HRV and conscious interoceptive attention	Participants were able to intentionally modulate blood pressure; HRV also improved. Findings suggest VR-assisted interoceptive/breathing biofeedback can produce state-level physiological regulation, although this is doctoral research rather than a peer-reviewed journal study.
Keskinkılıç, 2025	Adult participants (Master’s thesis)	Synchronous versus asynchronous avatar embodiment in VR	Interoceptive sensibility (THISQ) and embodiment	Avatar synchrony influenced embodiment, whereas trait interoceptive sensibility was not strongly associated with embodiment. Supports the importance of distinguishing state-dependent VR experiences from trait interoceptive measures.
Savickaite et al., 2026	General adult population (*N* = 26; predominantly neurotypical, with three ND participants)	Immersive VR with congruent versus incongruent motor cues	Cardiac interoceptive accuracy (heartbeat counting)	Motor congruency substantially increased body ownership. Interoceptive accuracy showed a trend-level positive association with body ownership during congruent movement, while alexithymia was negatively related to interoception. Highlights interactions between interoception, individual differences and embodiment.
Verbe et al., 2026	Neurologically healthy adults (*N* = 32; 19–45 years)	Immersive five-sided CAVE; heartbeat-counting performed with versus without passive social cues/virtual other	Cardiac interoceptive accuracy (heartbeat counting)	Cardiac interoceptive accuracy was significantly lower in the social VR condition. Demonstrates that seemingly minimal aspects of the virtual/social environment can alter interoceptive performance, potentially through competition between exteroceptive and interoceptive attention.
Ide-Okochi et al., 2022	School-aged children with developmental disabilities (*n* = 8) and typically developing controls (*n* = 7); DD group included neurodevelopmental conditions relevant to ASD/ADHD	VR classroom containing distracting events; gaze behavior and school-task performance assessed	Cardiac interoception (Heart Rate Perception test) and multidimensional interoceptive awareness (MAIA)	Children with developmental disabilities showed different gaze behavior, tended toward lower Heart Rate Perception performance and scored significantly lower on six of eight MAIA subscales. Demonstrates feasibility of combining VR, behavioral assessment and interoceptive measures in a neurodevelopmental population.
Döllinger, 2025	Series of adult VR studies presented within doctoral research	Manipulation of virtual embodiment/avatar representation, including virtual versus physical body and embodiment-related conditions	Body awareness/interoception	The programme of research indicates that virtual embodiment can influence awareness of internal bodily signals. Related empirical work found slightly reduced internal body awareness during virtual embodiment and showed that embodiment characteristics can mediate these effects. The dissertation synthesizes several studies rather than representing a single experiment.

El Ali et al. (2023) presents a combined screen/VR cardiac recognition paradigm with live ECG that manipulates the displayed heart rate and contrasts audible, visual, and audiovisual feedback. Accuracy did not differ between screen and VR, but adding audio increased interoceptive awareness (confidence), and, more importantly, presence linked adversely with cardiac accuracy, indicating that “being there” can compete with inward focus. The interviews were brief and strategy-oriented; the experienced phenomenology of interoception was not central. This tension between externally driven signals and internal bodily awareness echoes earlier experimental work demonstrating that cardio-visual synchrony can directly shape self-processing. For example, Suzuki et al., 2013 showed that synchronizing visual feedback with participants’ heartbeats modulates bodily self-consciousness, highlighting how interoceptive signals can be experimentally integrated into exteroceptive frameworks to alter embodiment.

A complementary clinical protocol in oncology frames VR as an intervention to cultivate interoception and body image through embodied scenarios, but as a protocol, it still relies on trait questionnaires (e.g., MAIA) and standard outcomes rather than in-VR state measures or qualitative co-interpretation of bodily experience (Sebri et al., 2023). Lived experience is recognized as motivation, not as data.

The two theses serve to bookend the design space. Torres et al. (2024) uses VR-assisted autonomic biofeedback (breathing + guided interoceptive attention) in spinal cord injury and controls, and observes cycle-wise blood-pressure modulation and heat rate variability increases during/after training; questionnaire interoception does not change, implying state-like rather than trait change. Importantly, the results appear to be driven by task design (breathing/inward focus) rather than display fidelity, which provides insight for developing VR activities that assess interoception rather than simply boosting presence. Keskinkılıç (2025) found that while interoceptive sensibility (THISQ) does not track embodiment, synchronous vs. asynchronous avatars did. The discussion openly advocates for larger experience notions (such as “embodied presence”) and state-sensitive measurements in VR.

More recently, Savickaite et al. (2026) examined cardiac interoception, alexithymia, and motor congruency in immersive VR. In a sample of 26 adults, congruent visuomotor feedback produced stronger body ownership than incongruent feedback, while individual differences in interoceptive accuracy and alexithymia were associated with aspects of embodiment. Although the sample was predominantly neurotypical, the findings are relevant to the present framework because they demonstrate that interoceptive ability and individual psychological characteristics may interact with experimentally manipulated sensorimotor information in shaping virtual body ownership. This further supports the need to measure variables such as alexithymia alongside interoceptive performance rather than treating responses to VR as technologically determined.

Verbe et al. (2026) provide further evidence that the virtual environment itself may alter interoceptive performance. Thirty-two neurologically healthy adults completed a heartbeat-counting task within an immersive CAVE under social and non-social conditions. Cardiac interoceptive accuracy was significantly reduced in the presence of passive social cues. The authors interpret this finding in terms of possible competition between interoceptive and exteroceptive attention. This is particularly relevant to the use of VR as an experimental testbed: social context is not necessarily a neutral feature of an immersive environment but may itself alter access to internal bodily signals. Consequently, variables such as avatar presence, social cues, attentional load, and experimenter context should be treated as potential experimental manipulations or confounds in future VR-interoception studies.

Importantly, preliminary work has also examined interoception within VR in neurodevelopmental populations. Ide-Okochi et al. (2022) assessed children with developmental disabilities and typically developing children using a virtual classroom containing distracting events. The study combined gaze behavior and task performance with the Heart Rate Perception test and the Multidimensional Assessment of Interoceptive Awareness (MAIA). Children with developmental disabilities showed differences in gaze behavior and significantly lower scores on several MAIA dimensions, alongside a tendency towards poorer cardiac interoceptive performance. Although this small feasibility study did not manipulate interoceptive inference directly, it demonstrates that VR-based assessment can be combined with interoceptive and behavioral measures in neurodevelopmental populations. It therefore qualifies the stronger claim that autism or neurodevelopmental conditions have not previously been examined in this context: rather, research directly manipulating interoceptive inference in immersive environments in autistic populations remains extremely limited.

A broader programme of work synthesized by Döllinger (2025) further examines the relationship between virtual embodiment, avatar representation, and awareness of the physical body. This doctoral research is useful for locating interoception within the wider VR embodiment literature and highlights the possibility that replacing or modifying the visually represented body may alter attention towards bodily signals. However, as this source is a doctoral dissertation synthesizing multiple studies rather than a single peer-reviewed experiment, its evidential status should be distinguished from the empirical journal studies discussed above.

These studies demonstrate that VR is already capable of manipulating attention, embodiment, and physiological feedback in ways that are directly relevant to interoceptive processing. Research directly manipulating interoceptive inference within immersive environments in autistic populations remains extremely limited. Although preliminary work has combined VR and interoceptive assessment in neurodevelopmental populations (Ide-Okochi et al., 2022), the existing literature remains dominated by non-autistic adult samples and clinical populations. The samples include anxiety patients, cancer survivors (planned), and students, highlighting a significant gap given how important interoception is in contemporary autism descriptions. The discipline remains cardio-centric (heart rate/heart rate variability; heartbeat tasks), with few respiratory/gastric tasks or metacognitive metrics included into VR.

Second, literature reflects a persistent technology-first focus. While head-mounted displays, tracking pipelines, physiological sensors, and standard metrics such as presence and simulator sickness (SSQ) are typically well specified, construct validity is often comparatively underdeveloped. In particular, many studies rely on trait-based measures of interoception (e.g., MAIA, THISQ), which are poorly suited to capturing the moment-to-moment, state-like nature of interoceptive access within immersive environments. This issue is compounded by the simplicity of experimental manipulations, which are frequently limited to binary contrasts (e.g., on/off or synchronous/asynchronous cardiac feedback), offering limited sensitivity to the complexity of interoceptive experience. Moreover, these manipulations are often weakly grounded in theory, with insufficient integration of frameworks such as predictive processing or multidimensional models of interoception, ultimately constraining both interpretation and cumulative progress in the field.

When state and trait are explored simultaneously, they double-dissociate: Torres et al. (2024) physiological indices move but trait scales do not; Breuninger’s (2017) subjective intensity rises while objective reactivity falls; El Ali et al.’s (2023) presence rises as cardiac accuracy declines. These are precisely the patterns that necessitate state measures that are consistent with the accuracy-sensibility-awareness framework, as well as frequent reporting of embodiment/presence as potential confounds rather than just attributes.

Third, lived experience is negligible. El Ali et al. (2023) conduct short, strategy-oriented interviews; the oncology protocol anticipates questionnaire changes; and the stress-induction and embodiment investigations use standardized measures. None incorporate micro-phenomenology or participatory co-design to guarantee that what is assessed corresponds to what participants mean by “feeling my heartbeat/breath/gut” in VR. Interpretability and translation are limited in a domain that is essentially personal and context-bound.

## From framework to testable experimental paradigms

The proposed framework is intended to generate falsifiable experimental predictions rather than provide only a descriptive account of interoceptive differences. One relatively direct implementation would manipulate cardio-visual synchrony within an embodied VR environment. Participants could view a subtle visual feature of an avatar, such as illumination around the chest, that is presented synchronously with the participant’s cardiac cycle, with parametrically increasing temporal delays, or independently of the heartbeat. Continuous ECG would provide the underlying cardiac signal, while interoceptive performance could be assessed using trial-wise synchrony judgements, confidence ratings and metacognitive correspondence between confidence and accuracy. Embodiment, presence, sensory load, anxiety and simulator symptoms should be measured concurrently rather than treated as incidental properties of the virtual environment.

A peripheral physiological model could estimate participant-specific cardiovascular state and regulatory parameters from the recorded physiology, while a separately specified perceptual model could estimate parameters describing the behavioral influence of prior expectations, sensory evidence and uncertainty. The critical prediction would not simply be that autistic and comparison participants differ in overall accuracy. Rather, individuals may differ in how strongly changes in cardio-visual reliability affect confidence and perceptual judgements, and these differences may be moderated by alexithymia, sensory profile and anxiety. Evidence against this interpretation would include equivalent sensitivity to manipulation across groups and individuals, or behavioral differences that disappear once physiological signal quality, sensory tolerability, attentional load and other person-level factors are controlled.

A second paradigm could examine respiratory interoception while moving beyond the field’s predominantly cardiac focus. Participants could experience a virtual environment in which visual or auditory cues representing breathing are gradually shifted away from the timing of their actual respiratory cycle. Alternatively, sensory load or social context could be manipulated while respiratory physiology remains continuously recorded. Participants would periodically report perceived breathing phase, respiratory effort, confidence and subjective bodily experience. This would allow researchers to ask whether contextual changes alter access to respiratory signals, whether changes arise primarily from altered physiology or perceptual interpretation, and whether state-level effects correspond to trait questionnaire measures.

These paradigms illustrate an important distinction within the proposed framework: VR manipulates the evidence and contextual conditions available for inference, peripheral modelling characterizes the bodily signal-generating process, and perceptual-inference modelling tests hypotheses about how observable responses might arise from integration of bodily evidence with expectations and context. None of these levels should be treated as interchangeable.

## Interoception as a computational problem

A critical missing element across current VR and physiological studies is the absence of lived experience as a formal component of interoceptive modelling. Autistic people frequently describe interoceptive experiences, such as delayed hunger, heightened heartbeat awareness under stress but not at rest, or difficulty distinguishing pain from anxiety, that do not map neatly onto canonical cardiac tasks or trait questionnaires (Adams et al., 2025; Itoi et al., 2022; Trevisan et al., 2021). These narratives help identify which bodily signals become salient, how they are interpreted, and which contextual factors shape access to them. From a computational perspective, they can inform model design by indicating which bodily modalities require attention, how precision weighting may vary across individuals, and where inference may break down or be compensated for. VR enables these lived experiences to be operationalized as controlled perturbations, such as distractor load, social context, or embodiment mismatches, while physiological modelling provides the internal signal landscape against which subjective reports and behavioral responses can be compared. In this way, lived experience becomes not merely anecdotal, but an empirical guide for mechanistic interoceptive science.

A key theme emerging from both autism research and VR studies is that interoception cannot be reduced to the raw detection of bodily signals. Contemporary theoretical frameworks, including predictive coding, active inference, and comparator models, suggest that interoception involves estimating hidden physiological states from noisy sensory evidence while integrating beliefs, context, and attention (Seth, 2013; Seth and Friston, 2016; Duquette, 2017). Behavioral performance in heartbeat or breathing tasks therefore reflects only the outcome of this inferential process, not the underlying computations. Similarly, although VR can precisely manipulate exteroceptive cues, embodiment, and attentional set, it does not by itself reveal how internal bodily signals such as heart rate, blood pressure, respiration, or autonomic regulation are changing during the task. Without modelling these bodily dynamics, it remains difficult to determine whether altered interoceptive performance reflects atypical physiology, atypical inference, or their interaction.

This is where computational physiological modelling becomes useful. Multiscale hemodynamic models can provide generative descriptions of the cardiovascular and respiratory dynamics that underlie interoceptive sensations (Li et al., 2026). In simple terms, VR can manipulate the perceptual and contextual conditions under which interoception occurs, while modelling helps characterize the bodily signals being inferred. Together, they offer a route toward a more mechanistic account of interoception in autism. The following section outlines the modelling foundations that make such an integrated approach possible.

## Interoceptive signals and computational modeling

Computational modelling offers a way to move from observable bodily signals to the latent physiological processes that generate them. Physiological signal-generation models refer to mechanistic models that describe how cardiovascular, respiratory, and autonomic states generate measurable bodily signals such as heart rate, blood pressure, and respiration. At the empirical level, bodily signals are directly observable physiological measures; however, these signals alone cannot determine whether altered bodily awareness reflects differences in baseline physiological state, signal variability, autonomic regulation, or precision weighting during inference (Chen et al., 2021; Petzschner et al., 2021). Perceptual-inference models, at the theoretical level, describe how an agent combines afferent bodily evidence with prior beliefs and contextual information to update its beliefs about bodily states. Generative mechanistic physiological models therefore play a complementary role by providing an explicit account of the bodily dynamics that participants are asked to perceive, interpret, or regulate. In future studies, estimated peripheral state trajectories and parameters from these models may constrain the likelihood mapping or expected state dynamics of a separately specified perceptual-inference model; however, they should not be treated as direct estimates of central prediction errors or precision weighting.

For autism research, this modelling perspective is particularly important. Existing interoceptive studies often rely on heartbeat tasks or trait questionnaires, yet autistic bodily experience is variable, context-dependent, and shaped by anxiety, alexithymia, attention, and sensory load. Computational physiological models can help bridge this gap by estimating latent peripheral physiological states and parameters that shape the bodily evidence available for interoceptive inference, such as baseline cardiovascular–respiratory state, response sensitivity, regulatory gain of autonomic control, and system-level coupling between physiological components. Lived-experience reports can further guide which bodily signals should be treated as interoceptively salient, for example whether subjective difficulty is associated with exaggerated challenge responses, delayed recovery, weak differentiation between autonomic modes, or context-dependent access to bodily signals. In future studies, when combined with separately specified perceptual-inference models and appropriate behavioral and subjective measures, these physiological models may help to test whether observed interoceptive patterns are associated with altered physiological dynamics, altered inference about bodily signals, or an interaction between the two.

Earlier zero-dimensional (0D) lumped-parameter and one-dimensional (1D) multiscale cardiovascular-respiratory models have provided a physical forward-model foundation for this approach. Individualized modelling frameworks developed by Li et al. (2022, 2023) demonstrate how hidden cardiovascular–respiratory dynamics can be estimated from observable physiological signals, thereby motivating the need to make autonomic control more explicit for interoceptive research. Simplified models of autonomic regulation have also been used in previous studies to simulate baroreflex mechanisms and their effects on cardiovascular control (Ottesen et al., 2004; Olufsen et al., 2006; Blanco et al., 2012). Building on this modeling line, Li et al. (2026) proposed a computational model that integrates the cardiovascular system (CVS), respiratory system (RS), and autonomic nervous system (ANS). With this specified model, heart rate and blood pressure are generated as outputs of interacting cardiovascular, respiratory, and autonomic mechanisms: the CVS provides the hemodynamic basis of signal generation, the RS captures respiratory modulation, and the ANS regulates these outputs through explicit sympathetic and parasympathetic control functions. By varying autonomic control parameters, the model can compare how different regulatory modes generate distinct cardiovascular–respiratory patterns across postural, physical, thermal, or cognitive challenges in typical-development and autism groups. These model-based comparisons make it possible to formulate testable hypotheses about not only whether heart rate or blood pressure differs between groups, but also which autonomic regulatory configurations may produce the bodily evidence available for interoceptive inference.

One hypothesis for future research is to connect these two modelling levels through a bidirectional but non-equivalent interface. First, peripheral physiological models could estimate participant-specific cardiovascular–respiratory state trajectories and regulatory parameters, such as baroreflex sensitivity and autonomic gain, from physiological signals recorded during controlled perturbations. These estimates could provide empirically grounded constraints on the range, temporal dynamics, and expected variability of bodily evidence supplied to the proposed central-level component—a separately specified perceptual-inference model, defined here as a probabilistic generative model of how beliefs about bodily states are estimated and updated from afferent evidence, prior beliefs, and contextual information. Petzschner et al. (2021) provide a conceptual basis for this division through a sensory–control loop in which internal states generate sensory signals, inference estimates those states, and regulatory actions alter subsequent internal states. Under a predictive-coding or active-inference implementation, the perceptual-inference model could operationalize this general function by specifying mappings between hidden bodily states and expected sensory observations, comparing predicted with observed bodily evidence, weighting the resulting prediction errors by their estimated precision, and updating posterior beliefs. An active-inference extension could additionally select regulatory policies through free-energy-based optimization (Allen et al., 2022). In the reverse direction, these regulatory policies could provide descending autonomic control inputs to the peripheral model, allowing future simulations of how belief-guided regulation alters subsequent physiology. This proposed coupling does not imply a one-to-one correspondence between parameters: baroreflex sensitivity and autonomic gain remain peripheral physiological parameters, whereas prediction errors, precision weighting, and belief updating are defined only within the separately specified perceptual-inference model and would require independent empirical validation.

Within this proposed interface, artificial intelligence (AI) could support, rather than define, the connection between peripheral physiological and perceptual-inference models. Multimodal inputs—including continuous heart rate and heart rate variability, beat-to-beat blood pressure, respiration, electrodermal activity, task and VR event markers, behavioral performance, state-sensitive self-reports, and contextual variables–could be used to estimate individualized peripheral states and regulatory parameters, characterize residual dynamics not captured by mechanistic equations, and derive testable constraints on the likelihood or state-transition structure of the separately specified perceptual-inference model. AI could also accelerate physiological simulations and provide real-time state estimates for adaptive VR perturbations. Its outputs should remain specific to the modelling level at which they are produced and should not be labelled as central prediction errors, precision estimates, or belief-update parameters without explicit representation and independent validation within the perceptual-inference model. Mechanistic components would be calibrated through constrained parameter estimation, whereas AI components would be developed using participant-level data and evaluated through participant-level cross-validation, held-out sessions, and, where possible, independent cohorts or tasks. Given the likely small and heterogeneous samples, future studies should use parsimonious inputs, safeguards against overfitting, transparent missing-data procedures, subgroup evaluation, and physiologically interpretable outputs.

## Toward a participatory, multimodal science of interoception in autism

Autistic individuals frequently describe interoceptive experiences that are variable, context-dependent, and difficult to interpret–such as delayed recognition of hunger or thirst, heightened awareness of cardiac signals under stress but not at rest, or difficulty distinguishing pain, anxiety, and other forms of affective arousal (Brewer et al., 2016; Shah et al., 2016; Livingston and Livingston, 2016; Suzman et al., 2021). These reports consistently indicate that access to bodily signals is neither uniform nor static, but shaped by attentional state, sensory load, predictability, and social context (Garfinkel et al., 2016; Nicholson et al., 2019). Importantly, such experiences do not map neatly onto canonical cardiac tasks or trait questionnaires, highlighting a disconnect between lived interoception and the constructs typically operationalized in experimental research (Shah et al., 2016; Hatfield et al., 2019; Loureiro et al., 2024).

From a computational perspective, these phenomenological accounts provide critical empirical constraints on models of interoceptive inference. They specify which bodily modalities are salient or ambiguous, where signal gain may be attenuated or amplified, and which contextual variables should modulate prior beliefs and precision weighting (Seth et al., 2012; Barrett and Simmons, 2015; Ainley et al., 2016). In this sense, lived experience is not anecdotal but foundational: it delineates the hypothesis space that generative models of interoception must respect if they are to meaningfully capture autistic bodily experience (Seth and Friston, 2016; Allen et al., 2022).

VR offers an experimental substrate for testing hypotheses about interoceptive inference. By systematically manipulating embodiment, exteroceptive load, agency cues, feedback modality and social context, immersive environments can alter the sensory evidence and contextual conditions under which bodily signals are interpreted. For example, changes in visual–proprioceptive synchrony can manipulate the reliability of body-related multisensory evidence, while cardiac or respiratory biofeedback can manipulate the correspondence between internal physiology and external cues (Gonzalez-Franco and Peck, 2018; El Ali et al., 2023). When combined with behavioral, metacognitive, physiological and appropriately specified computational measures, such manipulations can be used to test—rather than assume—whether observed effects are consistent with changes in prior expectations, sensory precision or belief updating.

Personalized peripheral models can estimate latent physiological states and regulatory parameters that are not directly observable from raw heart-rate, blood-pressure or respiratory signals alone. These may include cardiovascular–respiratory state trajectories, response sensitivity, recovery dynamics and parameters describing autonomic or baroreflex regulation. Such quantities characterize the bodily evidence available for interoceptive perception but should not be interpreted as neural prediction errors, precision estimates or belief-update parameters. Those constructs belong to a separately specified perceptual-inference model and would require behavioral, subjective and, where available, neural data for estimation and validation. Connecting the two levels is therefore a hypothesis to be tested: physiological model outputs may constrain the range and temporal structure of bodily evidence supplied to an inferential model, while the relationship between that evidence and perceptual judgements can be empirically estimated.

## Operationalizing participatory research

Participation can occur at several distinct levels and should not be conflated with the collection of qualitative data. Consultation involves seeking feedback on researcher-developed ideas, whereas co-design involves autistic collaborators contributing to the design of tasks, environments and measurement procedures. Co-production extends this involvement across research questions, interpretation and dissemination, while participant-led research places greater decision-making authority with autistic researchers or community partners. Separately, qualitative interviews or micro-phenomenological methods generate data about participants’ experiences but are not necessarily participatory unless participants also influence the research process.

Within the proposed framework, participatory input could occur before, during and after experimentation. During hypothesis generation, autistic collaborators could identify contexts in which bodily signals become more or less accessible and identify interoceptive modalities that matter in everyday life. During task development, collaborators could evaluate the interpretability, sensory tolerability and ecological relevance of VR manipulations. During analysis and interpretation, qualitative reports could be examined alongside behavioral and physiological data to determine whether model-derived distinctions correspond to participants’ descriptions of bodily experience.

For example, if autistic collaborators repeatedly report that heartbeat awareness becomes salient only during unpredictable social situations, this observation could motivate a VR design in which cardiac feedback reliability and social context are manipulated independently. The resulting experiment could test whether social context changes cardiac accuracy, confidence, physiological arousal, or the weighting of cardiac information in perceptual judgements. If physiological arousal changes while confidence and interoceptive performance remain stable, the qualitative account would not automatically be treated as evidence for altered precision; instead, it would motivate refinement of the task or alternative explanations. In this way, lived experience constrains the design and interpretation of models without being converted uncritically into computational parameters.

Participatory procedures should also accommodate different communication preferences through written, spoken, visual or asynchronous methods and should compensate community contributors appropriately. Where subjective reports, behavioral performance and physiological models disagree, these discrepancies should themselves be treated as informative rather than resolved by privileging one source of evidence. Such an approach reduces the risk that participation becomes tokenistic while preserving distinctions between experiential, behavioral, physiological and computational levels.

Autonomic regulation may represent an important contributor to individual differences in interoceptive experience in autism. Variation in cardiovascular and respiratory dynamics can alter the stability and predictability of the bodily signals available for interoceptive inference. Within predictive-processing accounts, less reliable bodily evidence could plausibly alter the relationship between interoceptive accuracy, subjective confidence and metacognitive awareness. Empirically, dissociations between dimensions of interoception have been reported in autistic adults, particularly in relation to anxiety (Garfinkel et al., 2016). Inconsistent selection of autonomic control modes has been linked to dissociations between interoceptive accuracy, sensibility, and metacognitive awareness, particularly under conditions of stress or uncertainty (Garfinkel et al., 2016). These dynamics suggest that autonomic dysregulation disrupts not only physiology, but the mapping between bodily states and subjective experience, with downstream consequences for emotion regulation, self-monitoring, and adaptive behavior. Computational modeling integrating the cardiovascular system, respiratory influences, and autonomic regulatory pathways provides an opportunity to infer internal regulatory mechanisms that are challenging to quantify directly and to better differentiate sympathetic and parasympathetic contributions to heart rate and blood pressure modulation.

Taken together, these considerations motivate a participatory, multimodal framework for interoception research in autism (Figure 1). In this framework, lived experience specifies what must be modelled; VR specifies how interoceptive inference can be altered in controlled yet meaningful ways; and computational physiological modelling specifies the latent mechanisms being perturbed. Measurement must be correspondingly multimodal, integrating objective physiology, behavioral performance, VR-specific metrics (e.g., embodiment, presence, attentional load), and state-sensitive phenomenological reports. The goal is not to collapse these levels into a single metric, but to triangulate inference across them, yielding personalized interoceptive profiles that reflect individual patterns of accuracy, sensibility, awareness, and precision weighting.

**FIGURE 1:**
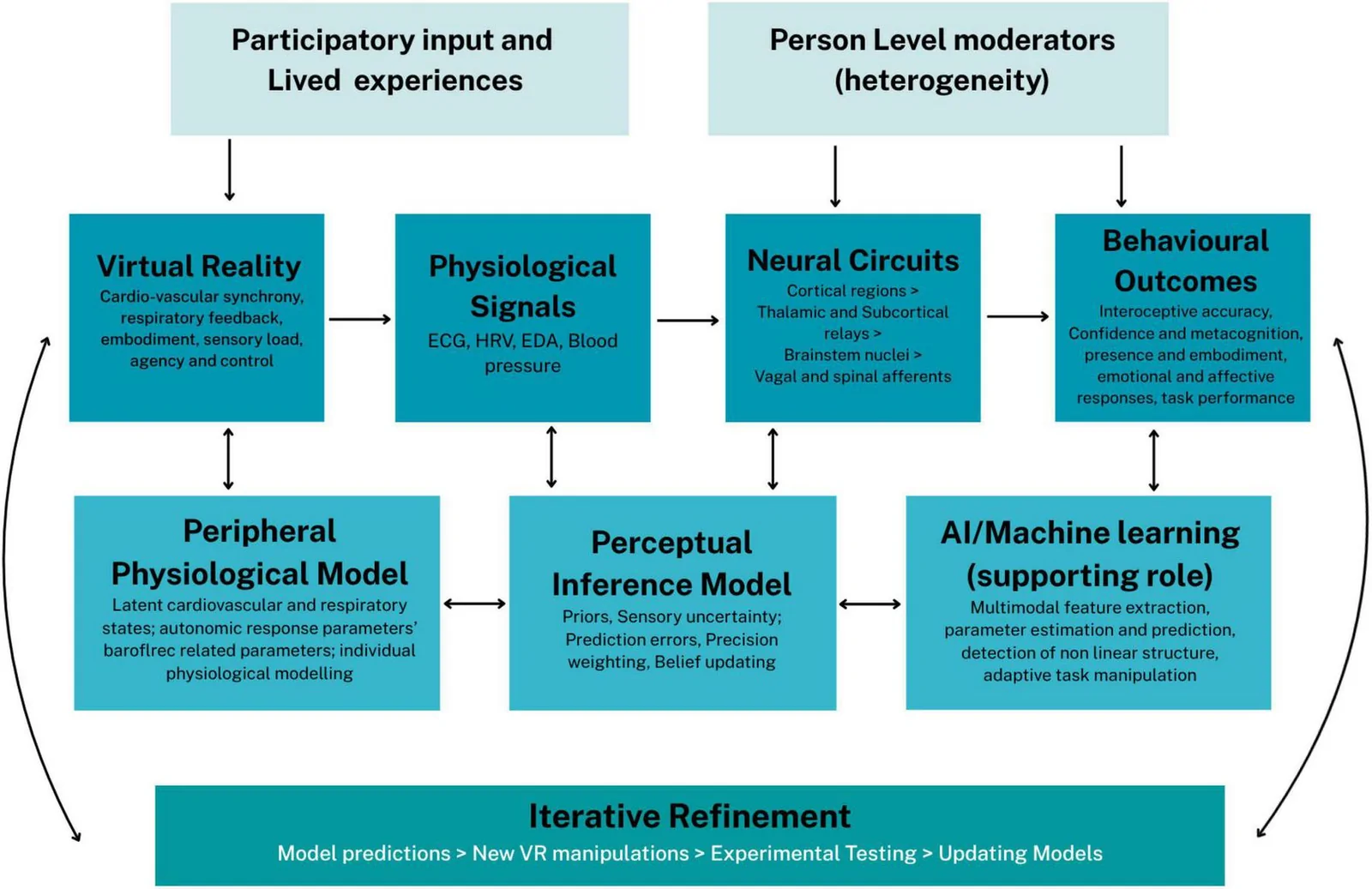
A multilevel framework for studying interoception in autism. Participatory input and lived experience inform the selection of relevant bodily modalities, contextual factors, and experimental manipulations, while person-level moderators capture heterogeneity in factors that may shape interoceptive processing. Virtual reality provides controlled manipulation of cardio-visual synchrony, respiratory feedback, embodiment, sensory load, agency, and related contextual variables, while physiological signals such as ECG, HRV, electrodermal activity, and blood pressure are recorded. These bodily signals are represented and regulated through distributed neural circuits spanning peripheral afferents, brainstem and subcortical relays, and cortical regions involved in interoception and salience processing. Behavioral and subjective outcomes include interoceptive accuracy, confidence and metacognition, embodiment, presence, affective responses, and task performance. Computational modelling is separated into peripheral physiological models, which estimate latent bodily and autonomic parameters, and perceptual-inference models, which formalize hypotheses concerning priors, sensory uncertainty, prediction error, precision weighting, and belief updating. AI and machine-learning methods are positioned as supporting tools for multimodal feature extraction, parameter estimation, prediction, and adaptive task manipulation. The framework is iterative, with model predictions informing new VR manipulations and experimental findings used to refine subsequent models.

Advancing this agenda requires several methodological commitments. First, lived experience must be treated as a primary data source, informing task design, modelling assumptions, and interpretation through micro-phenomenology, narrative elicitation, and co-production. Second, VR tasks should be designed around interoceptive inference rather than signal detection, extending beyond cardiac measures to respiratory, visceral, and multisensory domains. Third, personalized computational models should be employed to uncover latent mechanisms that behavioral tasks alone cannot resolve. Fourth, multi-method, preregistered designs should become standard, with explicit separation of accuracy, sensibility, and metacognition; systematic measurement of alexithymia, anxiety, and sensory sensitivity; and transparent reporting of VR-specific confounds. Finally, autistic people should be treated as co-theorists, contributing to the interpretation and validation of model outputs to ensure that computational constructs meaningfully reflect embodied experience.

## Limitations and practical challenges

The proposed framework also introduces substantial methodological and practical challenges. Most importantly, VR is not a neutral measurement environment. Presence, novelty, attentional capture, embodiment, cognitive load, social cues, demand characteristics and visual–vestibular conflict can independently alter subjective experience and autonomic physiology. The emerging literature reviewed above already provides evidence that characteristics of immersive environments can change measured cardiac interoception. Consequently, an observed change during VR cannot automatically be attributed to the intended interoceptive manipulation.

Future studies should therefore include appropriate control conditions. Depending on the research question, these may include non-immersive screen-based versions, matched immersive conditions without the critical interoceptive manipulation, or baseline conditions controlling sensory complexity and arousal. Presence, embodiment, simulator sickness, sensory discomfort and attentional load should be measured alongside interoceptive outcomes. Continuous physiological recording also creates its own reactivity problem: chest straps, electrodes, respiratory belts and other sensors may direct attention towards bodily signals and thereby change the construct being assessed. Familiarization periods, concealed or minimally salient sensors where appropriate, and matched sensor exposure across conditions can reduce—but not eliminate–this possibility.

Accessibility presents an additional challenge, particularly in autism research. Some participants may find head-mounted displays, headphones, restricted visual fields, controller demands, physiological sensors or unpredictable multisensory stimulation uncomfortable or inaccessible. Excluding people who cannot tolerate conventional immersive equipment would systematically narrow the population being studied. Flexible participation options should therefore be considered, including adjustable sensory intensity, seated modes, alternative interaction methods, shorter exposures, gradual familiarization, non-head-mounted immersive displays and matched non-immersive paradigms. Evidence from immersive assessment in other neurodevelopmental populations also indicates the value of individualized tolerability and alternative behavioral measures such as eye tracking (Fabio et al., 2026).

Cost and scalability are further limitations. High-quality head-mounted displays are increasingly affordable, but multimodal physiological recording, eye tracking, technical support, calibration and individualized modelling remain resource intensive. Data synchronization across VR events, physiology and behavioral responses introduces additional technical complexity, while mechanistic and machine-learning models may require data volumes that are difficult to obtain from small, heterogeneous participant groups. Multisite protocols, shared task specifications, preregistration and open analysis pipelines may improve reproducibility and scalability, but differences in hardware and recording systems will require careful calibration.

Finally, this framework should not be interpreted as assuming that more technologically complex measurement is inherently more valid. VR and computational modelling are useful only when they improve construct validity, permit theoretically meaningful manipulations, or distinguish explanations that simpler methods cannot resolve. Accessible behavioral tasks, qualitative methods and non-immersive experiments remain essential components of a multimethod approach.

## Conclusion

Ultimately, integrating lived experience with VR and computational modelling offers a route towards an interoception science that is both mechanistically informative and personally meaningful. Rather than treating autistic participants as deviations from neurotypical norms, a participatory framework recognizes lived descriptions of bodily experience as important evidence for identifying relevant modalities, contexts and hypotheses. VR can then experimentally manipulate these contexts, while physiological and perceptual-inference models provide complementary accounts of bodily dynamics and their interpretation. Triangulating phenomenological, behavioral and physiological evidence—rather than collapsing these levels into a single metric—may enable more individualized accounts of how autistic people perceive, interpret and regulate bodily states. This is essential for developing interventions and theories that respect neurodiversity and improve real-world wellbeing.
